# Editorial: Dysregulation of innate immunity in inflammatory skin disorders

**DOI:** 10.3389/fmed.2022.1024791

**Published:** 2022-09-26

**Authors:** Fang Wang, Christoph S. N. Klose, Jianzhong Zhang

**Affiliations:** ^1^Department of Dermatology, The First Affiliated Hospital, Sun Yat-sen University, Guangzhou, China; ^2^Department of Microbiology, Infectious Diseases, and Immunology, Charité—Universitätsmedizin Berlin, Freie Universität Berlin and Humboldt-Universität zu Berlin, Berlin, Germany; ^3^Department of Dermatology, Peking University People's Hospital, Beijing, China

**Keywords:** inflammation, Janus kinase, melanoma, autoimmune disease, atopic dermatitis

The skin is the largest organ in the body and the first to detect and combat various environmental stimuli such as toxins and pathogens (1). The skin has therefore developed a unique immune function, but this function also leaves it prone to immune dysregulation and various inflammatory conditions. Importantly, as an outer barrier, the skin is capable of reflecting internal pathologic conditions. Thus, an understanding of skin biology may lead us to make correct diagnoses of other internal diseases.

We are happy to see that this Research Topic involved a broad spectrum of skin conditions. For example, Feng et al. improved a murine model of atopic dermatitis (AD), a common allergic skin disease, and Xu et al. identified gene markers in cutaneous melanoma (CM) that are related to immune responses like pyroptosis and immune infiltration. Further study of the multi-faceted biology of skin may give us a deeper understanding of inflammatory skin conditions.

AD is a chronic and relapsing skin disorder characterized by extensive itching and inflammatory skin lesions (2). Its debilitating symptoms severely impair patients' quality of life. AD is predominated by type 2 inflammation and the production of allergen-specific IgE. Murine models that can show both inflammation and allergen responses are therefore of significant importance to AD research (3, 4). On this topic, Feng et al. improved an AD murine model by applying dinitrofluorobenzene, a common hapten, in addition to an extract of *Dermatophagoides farinae*, an allergen, to the murine back skin. They verified that the skin lesions and scratching behavior seen in AD were reproduced by this model. Furthermore, a type 2 immune profile was also shown. This model therefore provides another useful tool for AD research.

Nowadays, Janus kinase (JAK) inhibitors have been approved worldwide for the treatment of AD (5, 6). However, given that substances closely related to JAK are involved in various pathological processes, JAK inhibitors are often prescribed off-label (7). Diffuse cutaneous systemic sclerosis (SSc) is a connective tissue disease that is lacking in satisfying treatment options. Hou et al. recruited 10 adult patients with SSc and treated them with the JAK inhibitor baricitinib. Strikingly, the symptoms of skin fibrosis and digital ulcers were significantly improved at week 12 compared with baseline, and even more improved at week 24. The authors further employed a bleomycin-induced murine model of SSc and found that a JAK2 inhibitor can significantly reduce dermal thickening and collagen accumulation in this setting. Taken together, this study provides clinical and experimental evidence of the efficacy of JAK inhibitors in treating sclerosing skin diseases.

Autoimmune diseases, such as lupus, usually present skin symptoms. Indeed, even in some uncommon autoimmune conditions such as Still's disease and pyoderma gangrenosum, acne, and hidradenitis suppurativa (PASH) syndrome, the skin becomes either a diagnostic clue or a site of predilection for the condition. Rao et al. reviewed recent findings on the clinical manifestations, mechanisms, laboratory examinations, and treatment approaches of adult-onset Still's disease (AOSD). They found that T cell activation and proliferation are usually exhibited by patients with AOSD. As the skin lesions are often atypical and therefore not reliable for correct diagnosis, histopathologic examination may help. To date, treatment plans for AOSD are based on sporadic cases, retrospective case studies, and small clinical trials (8). Although traditional anti-inflammatory drugs are still considered first-line therapies in this context, biologic medications such as tumor necrosis factor alpha (TNF-α) inhibitors and interleukin (IL)-1β inhibitors have also been found to exhibit efficacy for AOSD.

PASH syndrome is a rare autoinflammatory condition that mainly occurs in the skin and has typical skin manifestations (9). Huang et al. presented a case study of a young Chinese man with PASH syndrome. Although the patient initially responded well to systemic steroid treatment, the disease recurred when the steroid was tapered. The authors then prescribed thalidomide, colchicine, and doxycycline, in addition to prednisone, and this combination of therapies provided significant relief. They concluded that, although traditional immunosuppressants can be effective for PASH syndrome, emerging biologic medications are worthwhile to try, especially in future clinical trials.

Melanoma is a potentially lethal malignant tumor. Although several therapeutic approaches, such as immunotherapies, targeted therapies, and chemotherapies, have shown efficacy in controlling this disease (10), biomarkers and prognostic models are still needed to improve disease prognosis. Xu et al. started from the pyroptosis-triggered inflammatory response, which contributes to this disease's distinct tumor microenvironment. They collected and analyzed gene expression profiles from the database of The Cancer Genome Atlas (TCGA) and constructed a gene signature that comprises genes related to inflammation and pyroptosis (GRIPs). They also verified their findings *via* real-time quantitative PCR. Ultimately, they concluded that the risk of poor prognosis may be related to pyroptosis and regulation of immune responses, such as T cell activation. These findings not only indicate biomarkers for melanoma but also provide potential targets for future therapeutics.

In summary, this Research Topic contains studies of several different kinds that investigate skin function as well as various skin diseases, their pathogenesis, and potential treatments. We believe that these articles are worthwhile to read and will provoke interesting ideas for future studies.

## Author contributions

FW, CK, and JZ designed the article structure. FW wrote the manuscript. CK edited the language. All authors contributed to the article and approved the submitted version.

## Funding

FW was funded by the Ministry of Science and Technology of the People's Republic of China (2022ZD0206200) and the National Natural Science Foundation of China (Grant No. 82073427). CK was supported by grants from the European Research Council Starting Grant (ERCEA; 803087 to CK) and the German Research Foundation (DFG; Project-ID 259373024—CRC/TRR 167, B05—CRC/TRR 241, FOR2599 project 5—KL 2963/5-2, SPP1937—KL 2963/2-1, and KL 2963/3-1 to CK).

## Conflict of interest

The authors declare that the research was conducted in the absence of any commercial or financial relationships that could be construed as a potential conflict of interest.

## Publisher's note

All claims expressed in this article are solely those of the authors and do not necessarily represent those of their affiliated organizations, or those of the publisher, the editors and the reviewers. Any product that may be evaluated in this article, or claim that may be made by its manufacturer, is not guaranteed or endorsed by the publisher.
